# Supplementary figures and images for: Differential gene expression analysis and cytological evidence reveal a sexual stage of an amoeba with multiparental cellular and nuclear fusion
Source: PLoS One. 2020 Nov 4;15(11):e0235725. doi: 10.1371/journal.pone.0235725 (PMC7641356; doi:10.1371/journal.pone.0235725)

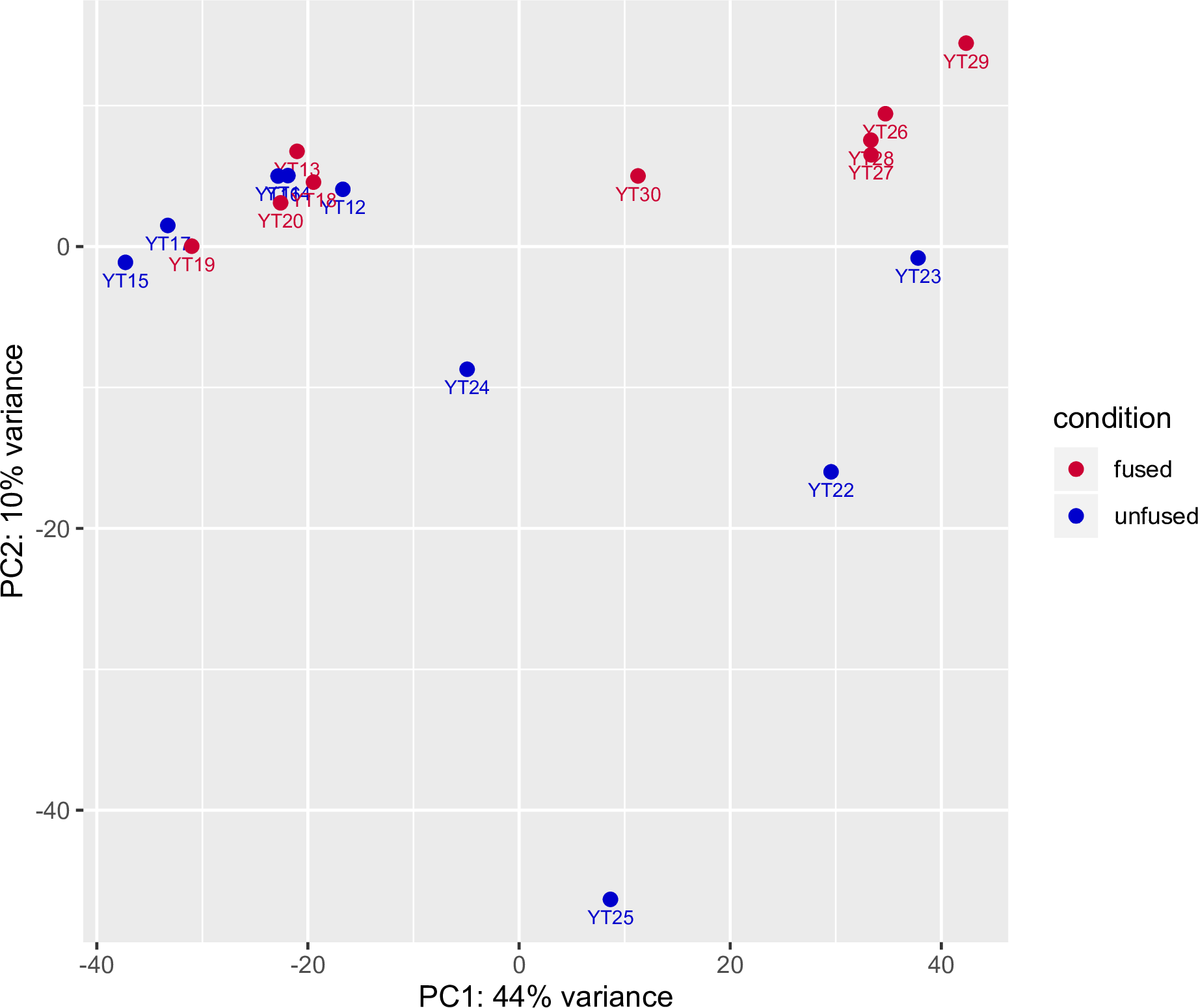

Supplement: S1 Fig — PCA data for the plot were the transformed normalized counts of each sample generated from DESeq2. (TIF) [file pone.0235725.s001.tif]

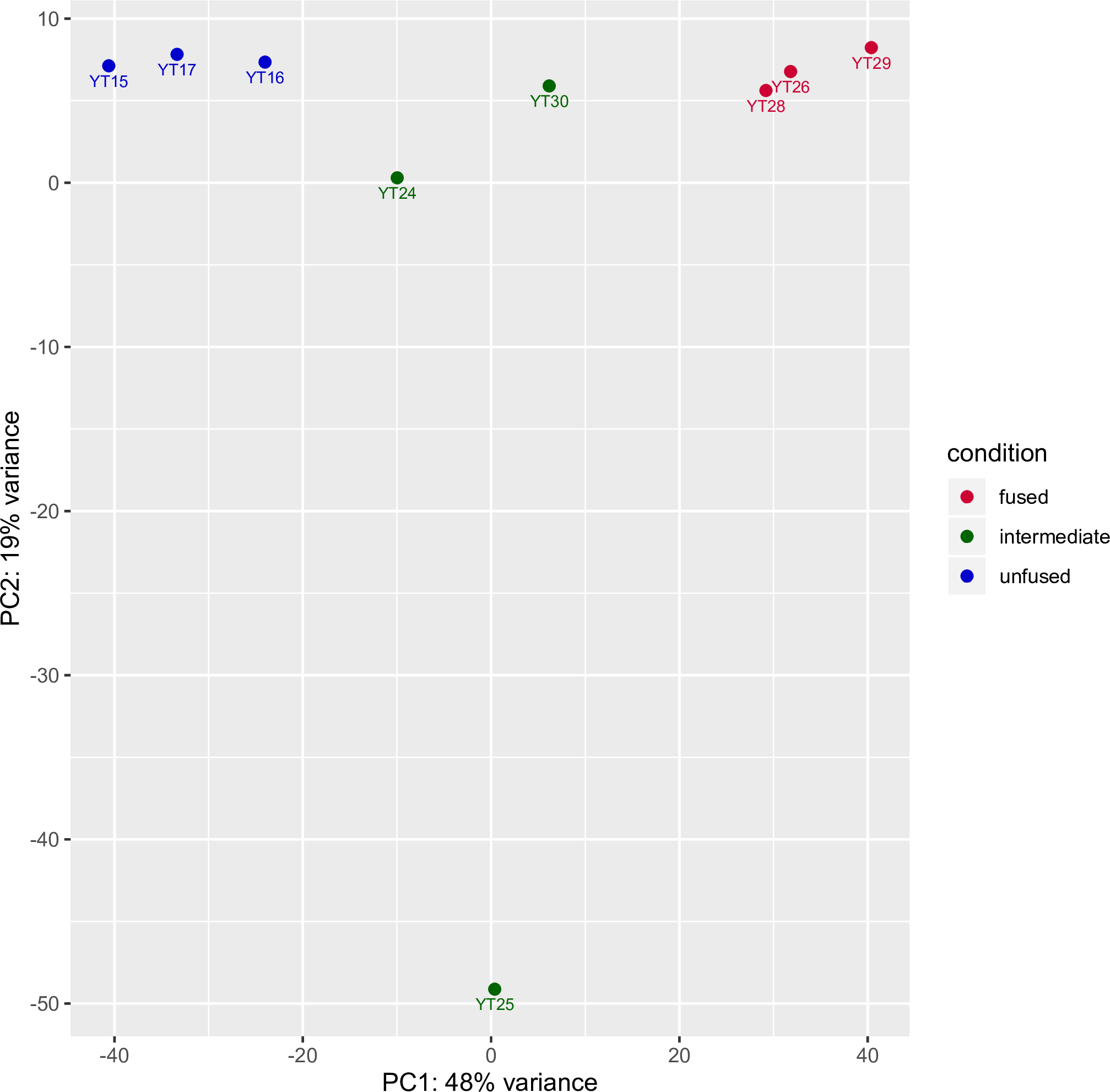

Supplement: S2 Fig — (TIF) [file pone.0235725.s002.tif]

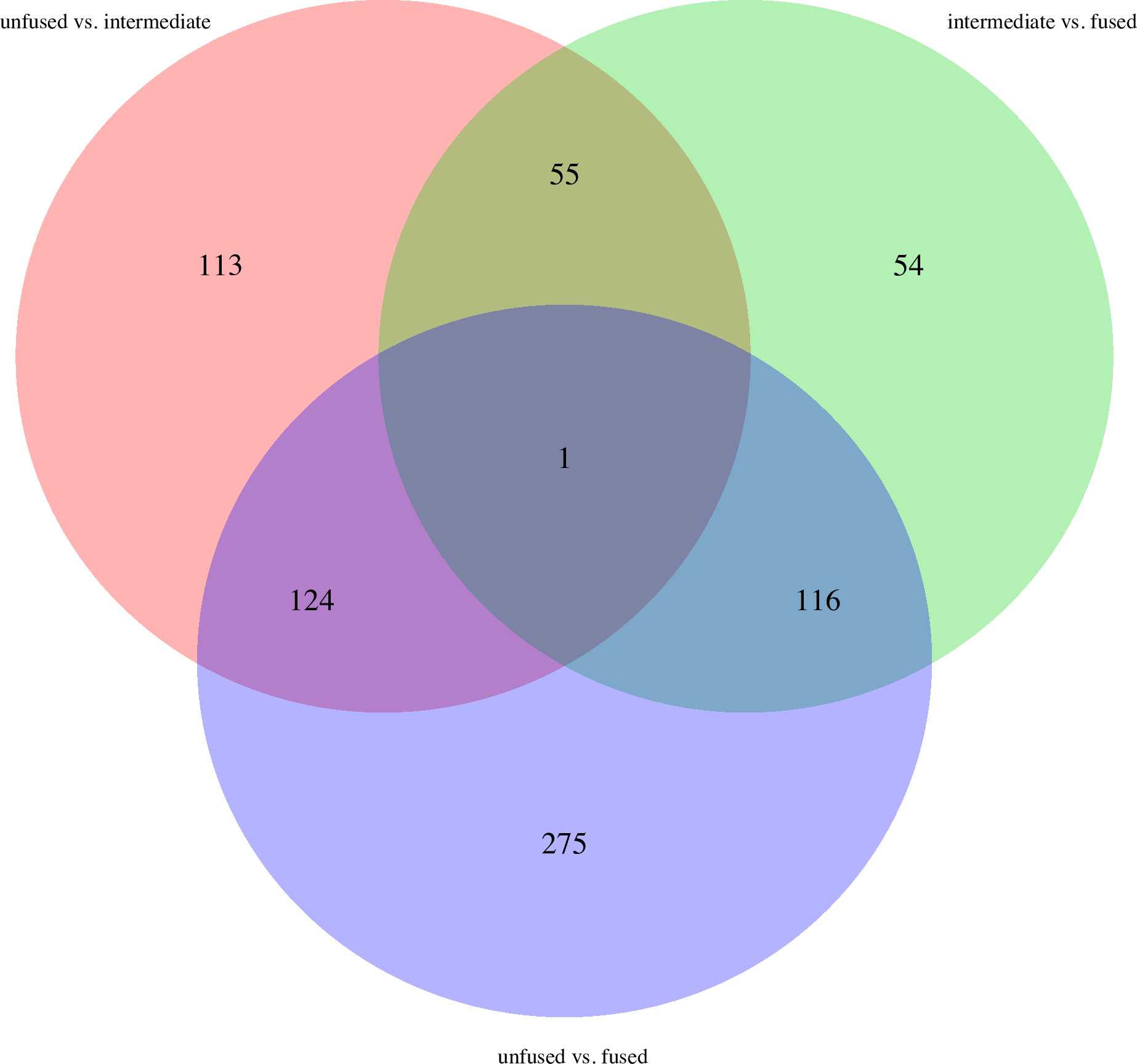

Supplement: S3 Fig — (TIF) [file pone.0235725.s003.tif]

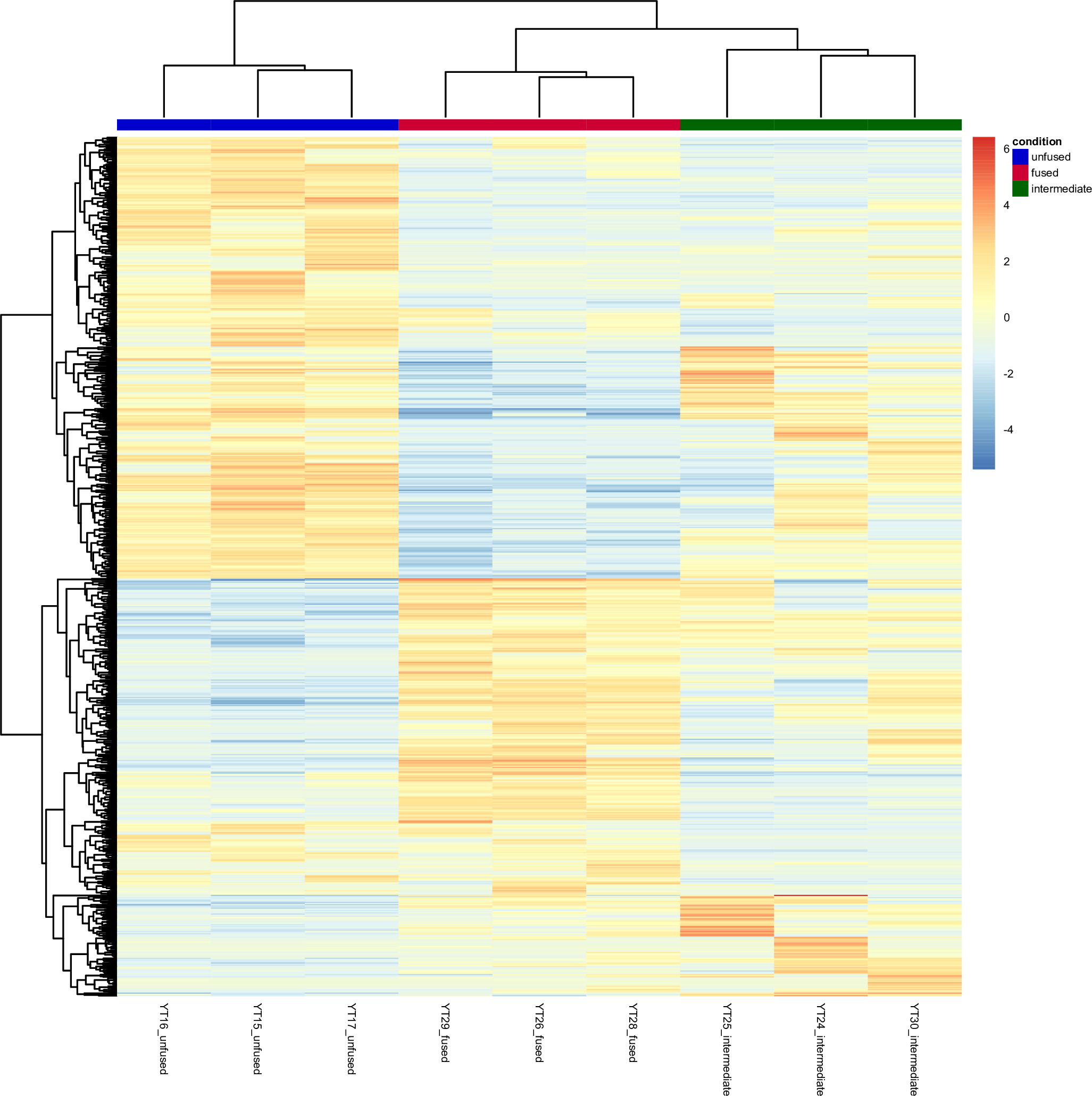

Supplement: S4 Fig — The color scale from red (highly expressed) to blue (low expression) represents the transformed, normalized counts from variance stabilizing transformation. (TIF) [file pone.0235725.s004.tif]

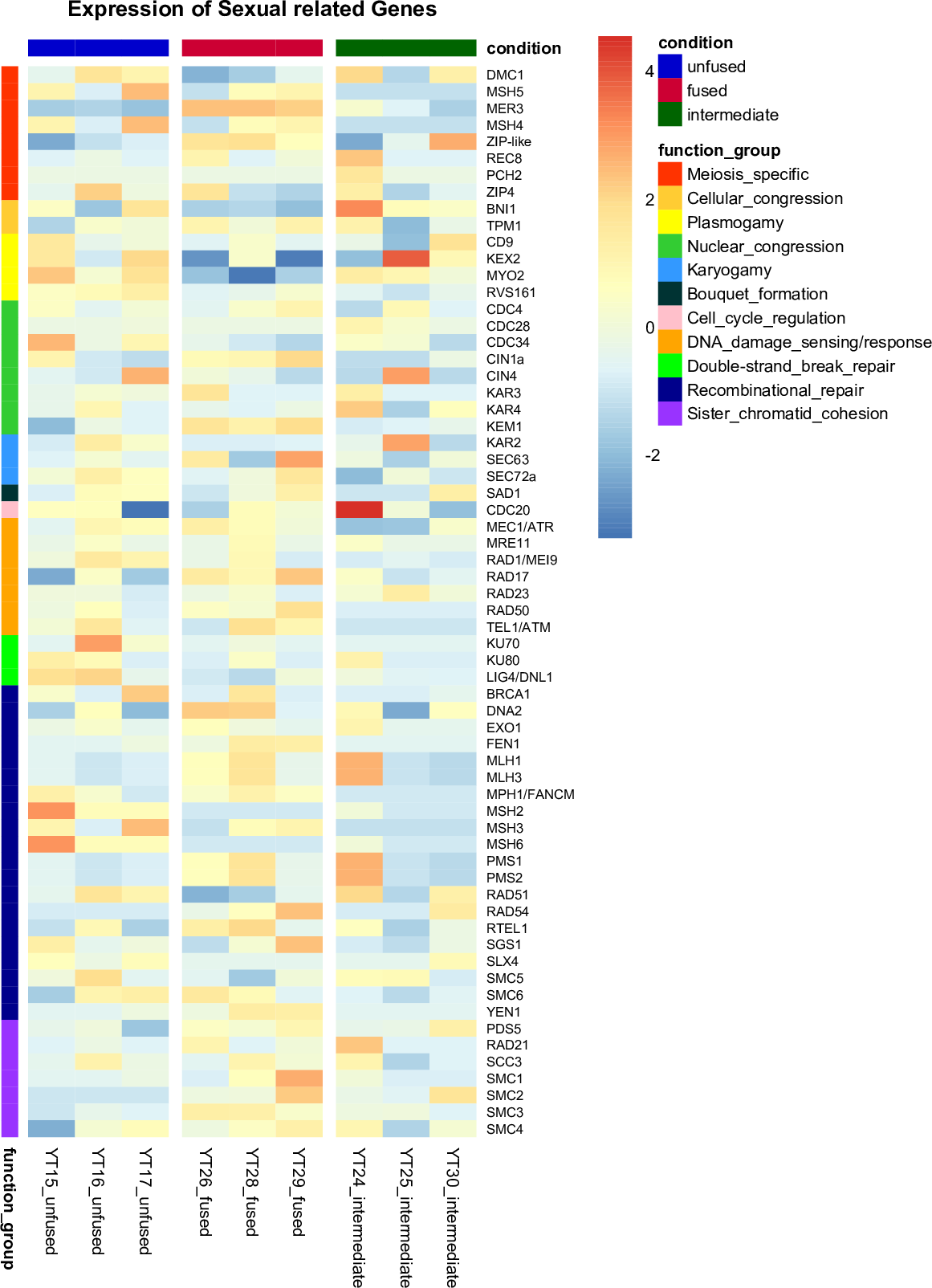

Supplement: S5 Fig — The heatmap was generated by centering the values across samples, and thus shows the deviation of each gene in each sample using the data set from the DESeq2 package. The color scale from red (highly expressed) to blue (low expression) represents the transformed, normalized counts from variance stabilizing transformation. Function categories were shown in the annotation bar. (TIF) [file pone.0235725.s005.tif]

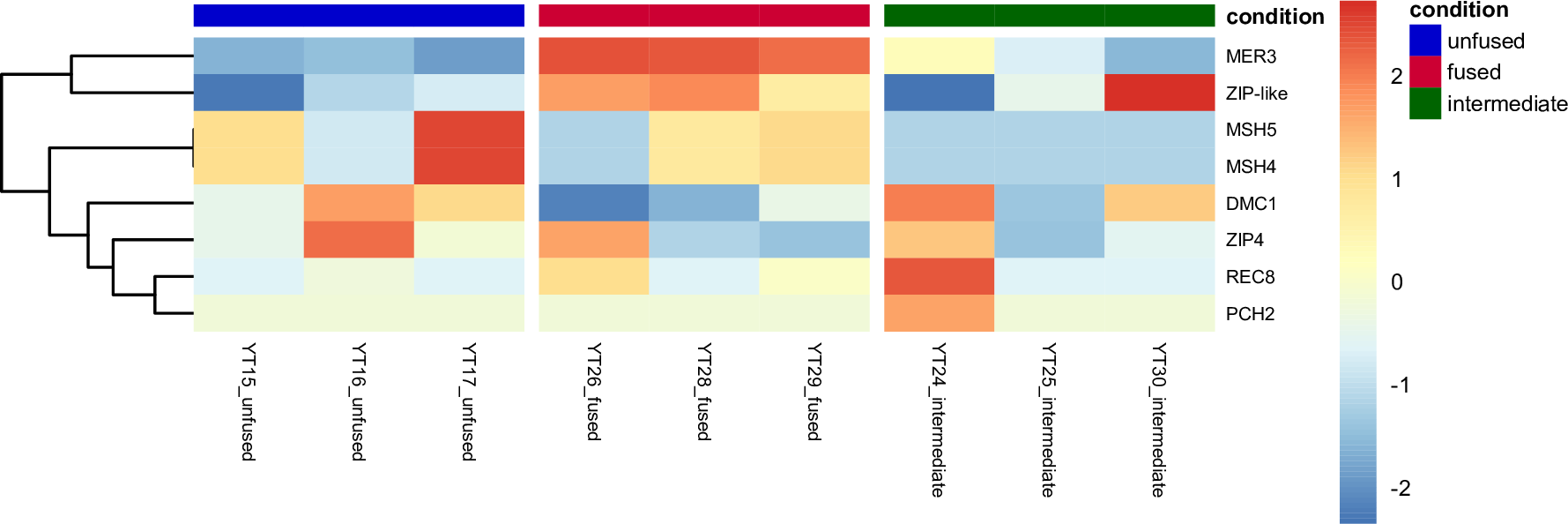

Supplement: S6 Fig — (TIF) [file pone.0235725.s006.tif]

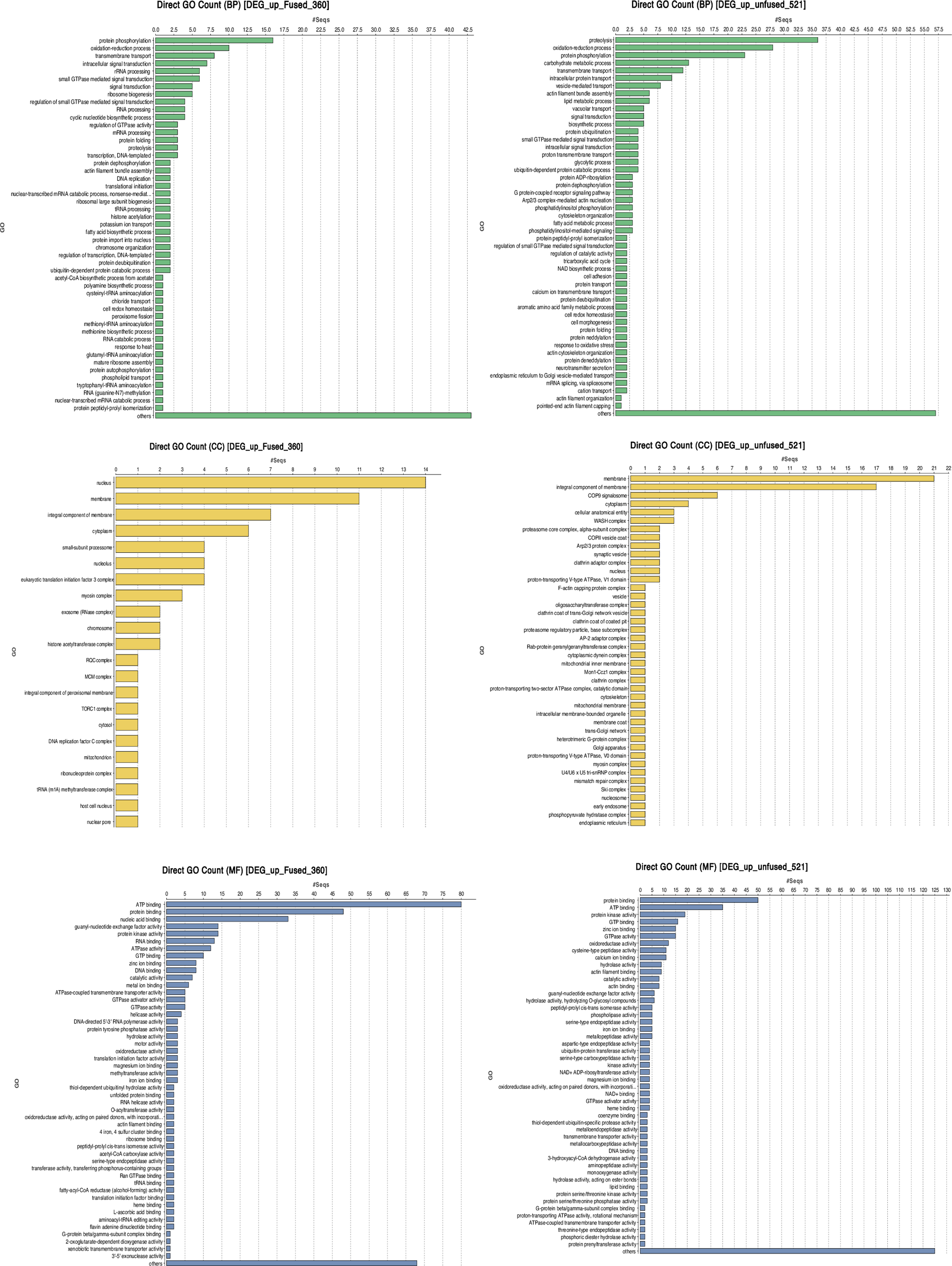

Supplement: S7 Fig — The plots were generated from Blast2GO without taking account of the GO hierarchy. The left panel shows the results for 360 DEGs upregulated in fused samples and the right panel shows the results for 521 DEGs upregulated in unfused samples. (TIF) [file pone.0235725.s007.tif]

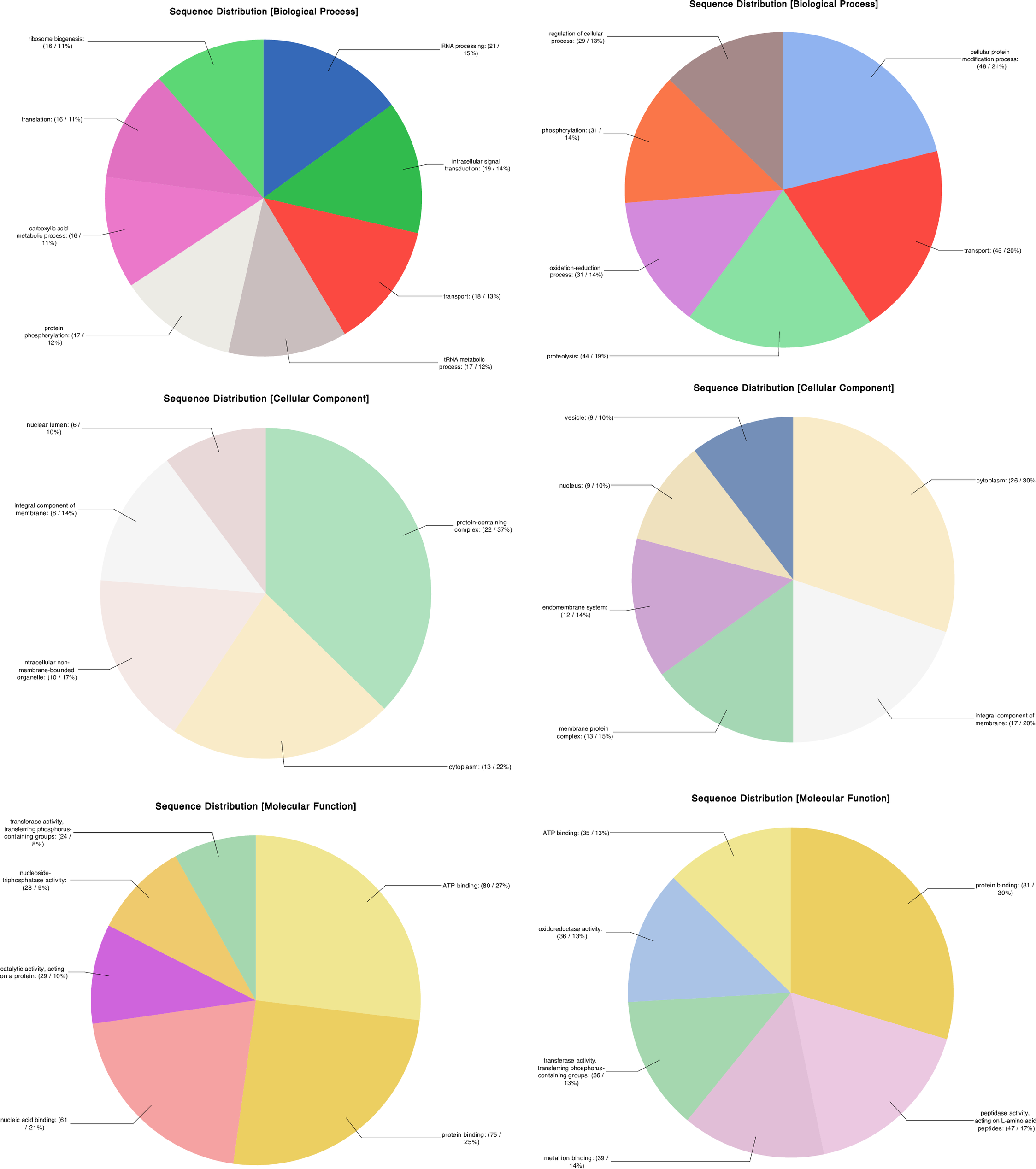

Supplement: S8 Fig — These were generated with the lowest node per branch of the combined GO graph from Blast2GO for each of the three GO categories (Molecular function, Biological process, Cellular components) with sequence number and percentage. The left panel shows the results for 360 DEGs upregulated in fused samples and the right panel shows the results for 521 DEGs upregulated in unfused samples. (TIF) [file pone.0235725.s008.tif]

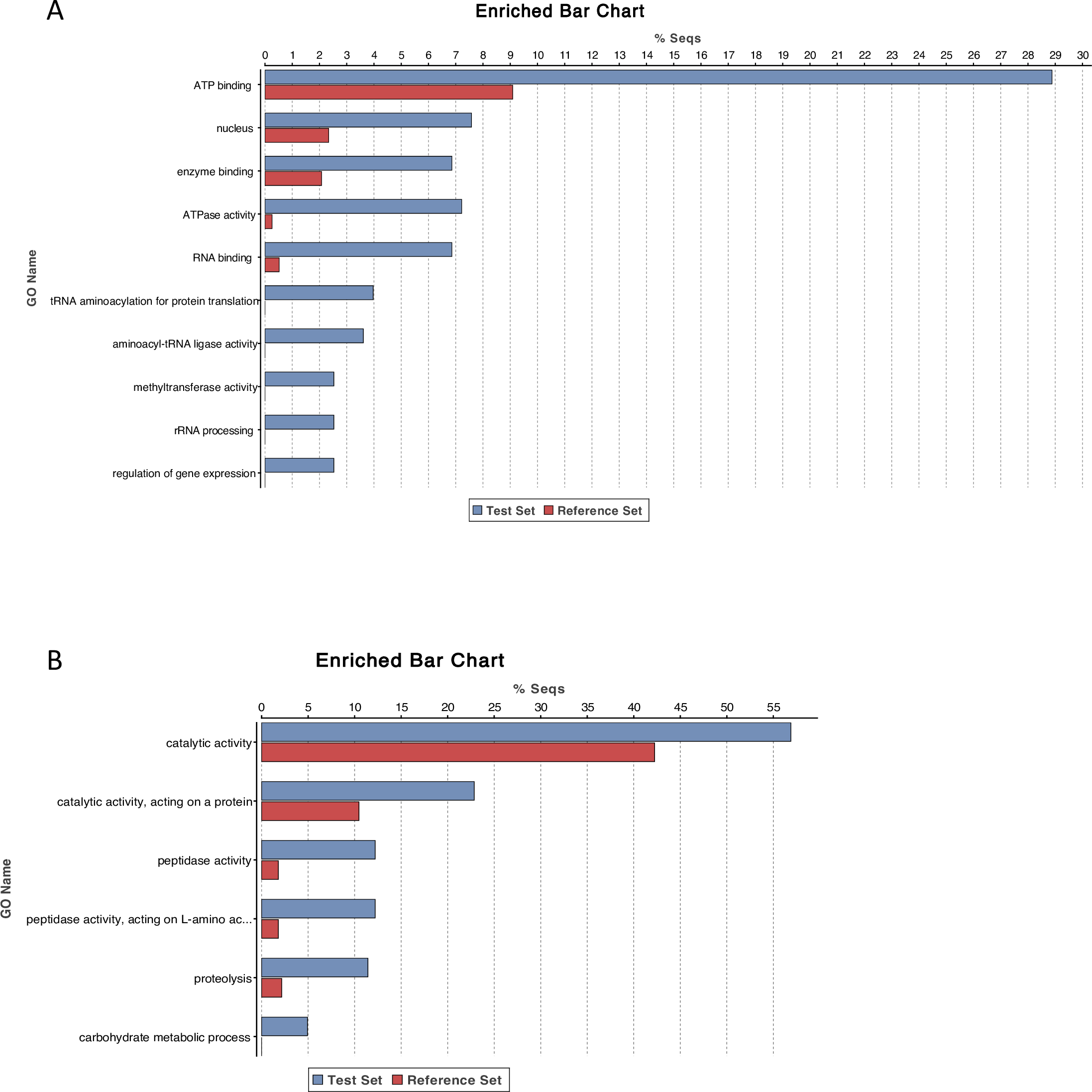

Supplement: S9 Fig — (A) The test gene set was for the 360 DEGs upregulated in fused samples. (B) The test gene set was for the 521 DEGs upregulated in unfused samples. In both tests, all 881 DEGs were set as the reference set. (TIF) [file pone.0235725.s009.tif]

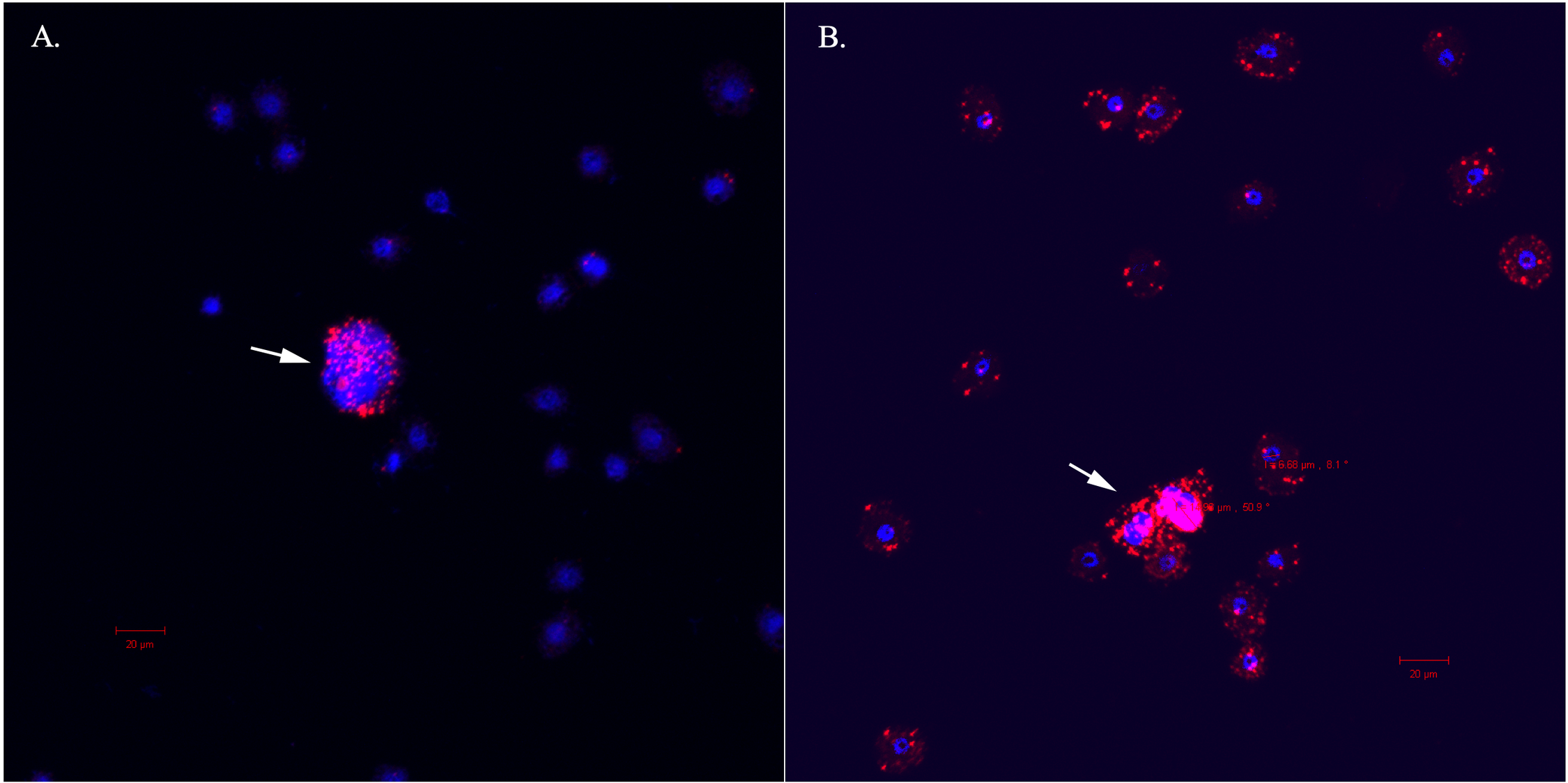

Supplement: S10 Fig — Note the variation in expression levels among single uninucleate cells. In (A) single cells seem to be expressing less Mer3 than single cells in (B). Mer3 is consistently expressed in higher quantity in fused cells (A and B, arrows). Red (Mer3) and Blue (DNA). Scale bar = 10 μm. (TIF) [file pone.0235725.s010.tif]

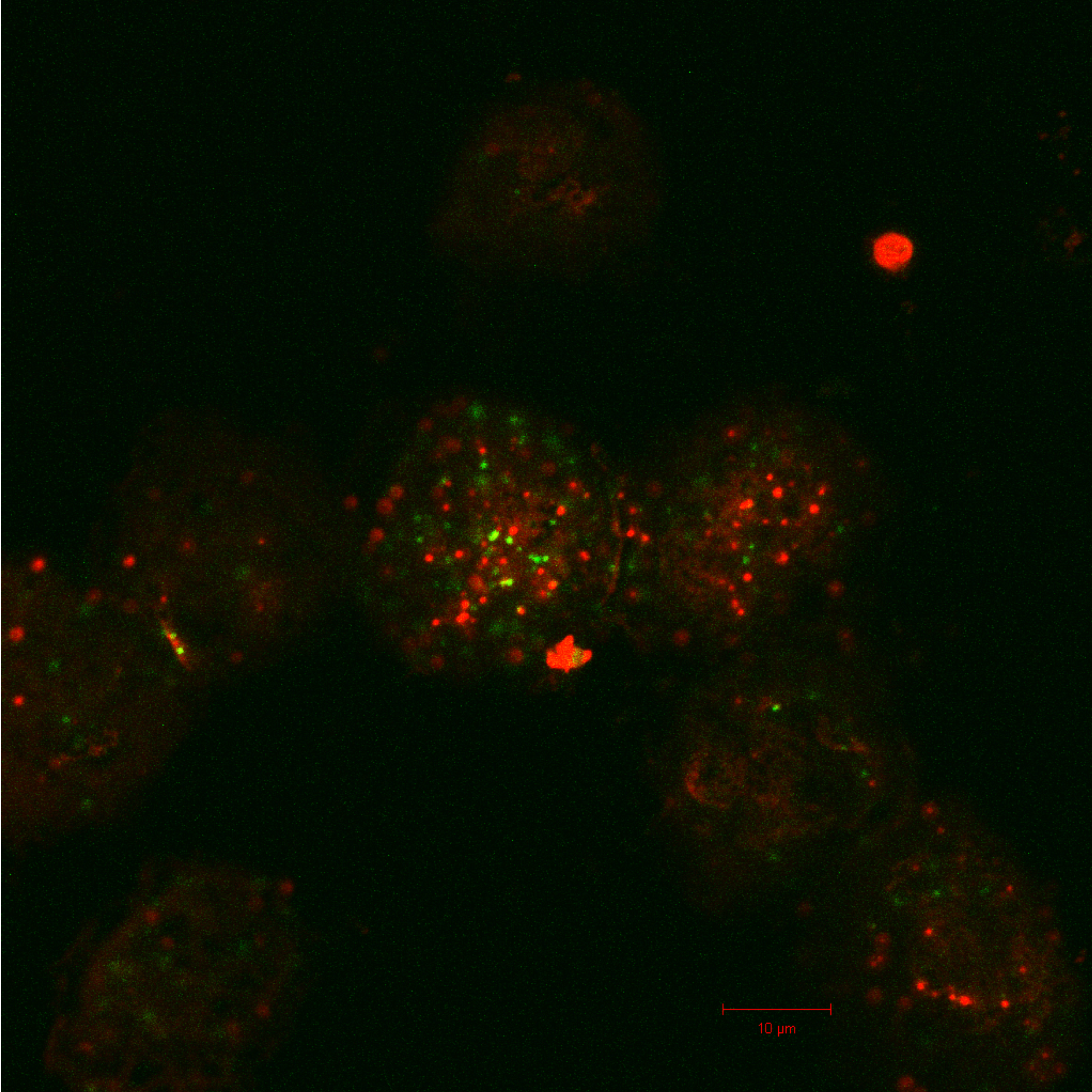

Supplement: S11 Fig — (TIF) [file pone.0235725.s011.tif]

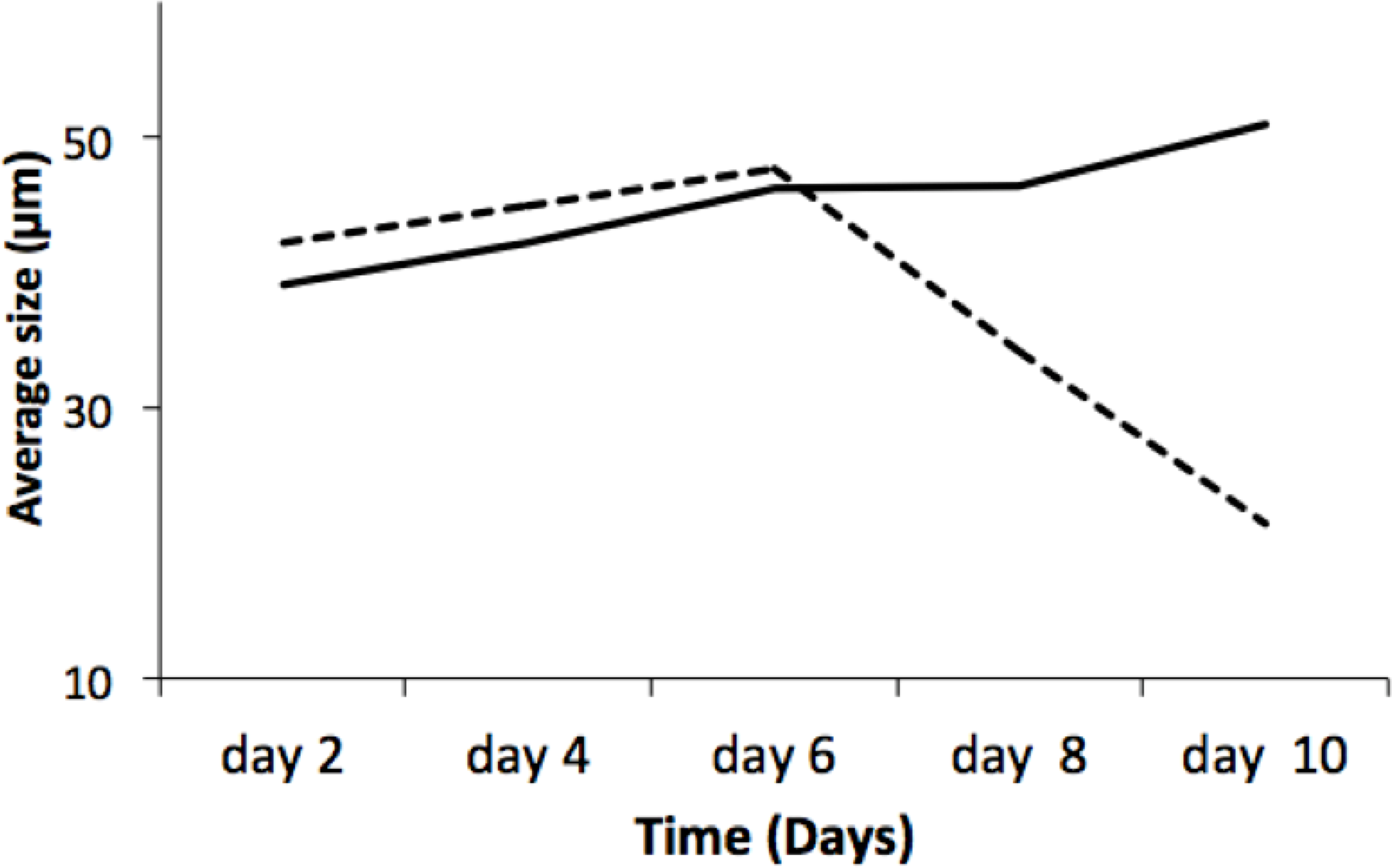

Supplement: S12 Fig — (TIF) [file pone.0235725.s012.tif]
